# Supplementary material for: Engineering microalgal cell wall-anchored proteins using GP1 PPSPX motifs and releasing with intein-mediated fusion
Source: bioRxiv. 2025 Jul 8:2025.01.23.634604. Originally published 2025 Jan 24. Preprint. [Version 2] doi: 10.1101/2025.01.23.634604 (PMC11785195; doi:10.1101/2025.01.23.634604)
Supplement: Supplement 1 [file NIHPP2025.01.23.634604v2-supplement-1.pdf]

bioRxiv preprint doi: https://doi.org/10.1101/2025.01.23.634604; this version posted July 8, 2025. The copyright holder for this preprint (which was not certified by peer review) is the author/funder, who has granted bioRxiv a license to display the preprint in perpetuity. It is made available under aCC-BY 4.0 International license.

Supplementary Figures

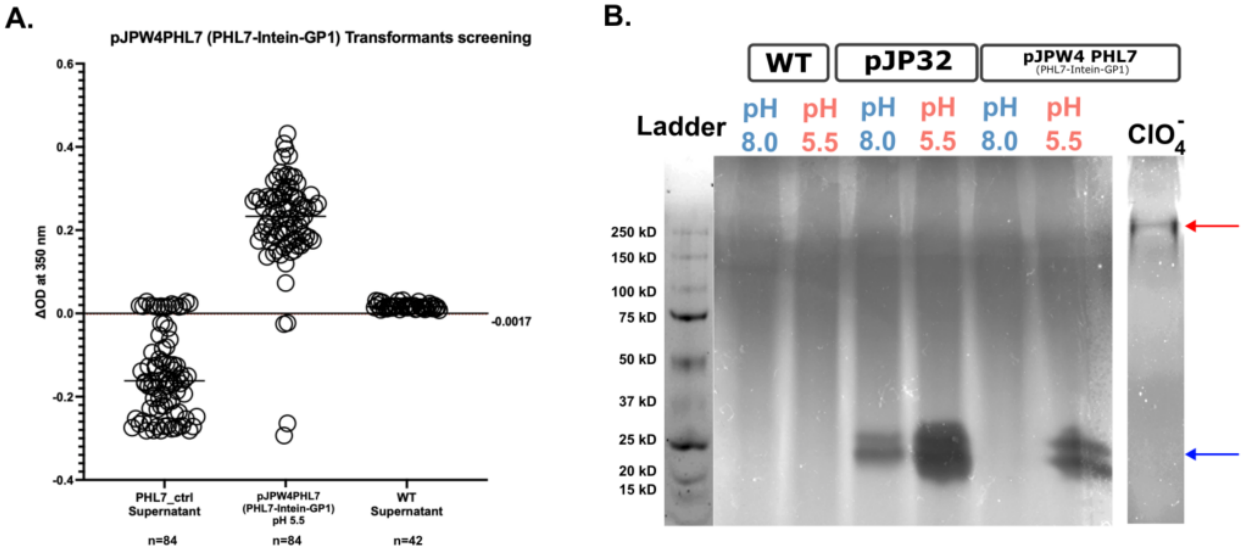

Supplementary Figure 1: Zymogram analysis of plastic-degrading activity in *C.reinhardtii* strains expressing PHL7 under different pH conditions and perchlorate treatment. **(A)** Impranil® activity assay to screen for positive PHL7-intein-GP1 transformants. The threshold (red dotted line) for positive transformants represents three standard deviations below the mean WT supernatant activity (-0.0017). **(B)** This zymogram, performed using Impranil®, a plastic dispersion substrate, detects plastic degrading activity of the plastic-degrading enzyme PHL7 in samples collected from wild type (*WT*), pJP32 (PHL7-secreting top expressing strain), and PHL7-intein-GP1 (PHL7-intein-GP1 fusion protein-top expressing strain). The WT strain exhibited no detectable activity at either pH 8.0 or pH 5.5. The pJP32 strain, which secretes PHL7, showed clear plastic degrading activity at both pH 8.0 and pH 5.5, indicating functional secretion of the plastic-degrading enzyme. In contrast, the PHL7-intein-GP1 top expressor displayed no detectable activity at pH 8.0, but strong plastic degrading activity was observed at pH 5.5, consistent with pH-dependent cleavage and release of the active enzyme from the GP1-Intein-PHL7 fusion construct. Additionally, treatment of the PHL7-intein-GP1 strain with perchlorate (ClO□□) resulted in a clearing zone at above 250 kD (red arrow), indicating an initially uncleaved form of the protein, in contrast to the expected ~25 kDa for the free form (blue arrow). This result highlights the pH- and chemical-dependent regulation of PHL7 release and activity in engineered *C. reinhardtii* strains.

bioRxiv preprint doi: https://doi.org/10.1101/2025.01.23.634604; this version posted July 8, 2025. The copyright holder for this preprint (which was not certified by peer review) is the author/funder, who has granted bioRxiv a license to display the preprint in perpetuity. It is made available under aCC-BY 4.0 International license.

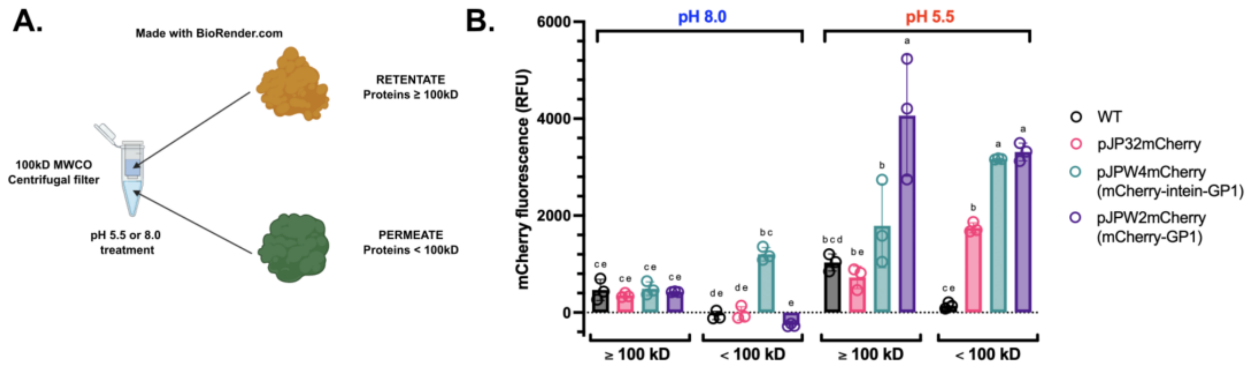

Supplementary Figure 2: Analysis of mCherry fluorescence in supernatants of *C. reinhardtii* strains treated at pH 8.0 and pH 5.5 and filtered through a 100 kDa molecular weight cutoff (MWCO) filter.

(A) Schematic showing a nanofiltration-based fractionation assay using a 100 kDa molecular weight cutoff filter (MWCO), which allowed us to separate samples into retentate (≥100 kDa) and flowthrough (<100 kDa) fractions. Made with BioRender.com. **(B)** The bar plots show mCherry fluorescence, measured in relative fluorescence units (RFU), in two filtrate fractions: proteins smaller than 100 kDa ("<100 kDa") and retained proteins larger than 100 kDa (">100 kDa"). Samples were analyzed from wild type (WT), pJP32 (secreting non-fused mCherry), mCherry-GP1 (top expressor), and mCherry-intein-GP1 (top expressor). **pH 8.0 (left panel)**: Low fluorescence was detected in wild-type, pJP32 *and* mCherry-GP1 fractions across both MWCO filters fractions. mCherry-intein-GP1 strains demonstrated higher fluorescence in the <100 kDa fraction, suggesting partial release of mCherry-containing proteins under neutral conditions. **pH 5.5 (right panel):** Acidic treatment resulted in a substantial increase in fluorescence in the <100 kDa fraction for all recombinant strains, pJP32, mCherry-GP1 and mCherry-intein-GP1, suggesting pH-dependent release of mCherry. pJP32 exhibited modest fluorescence in the <100 kDa fraction, consistent with secretion of mCherry. The >100 kDa fraction fluorescence decreased significantly, particularly for mCherry-intein-GP1, indicating potential dissociation or cleavage of the fusion protein under acidic conditions. Letters above bars indicate statistical groups, with distinct letters denoting significant differences ($p < 0.05$).

bioRxiv preprint doi: https://doi.org/10.1101/2025.01.23.634604; this version posted July 8, 2025. The copyright holder for this preprint (which was not certified by peer review) is the author/funder, who has granted bioRxiv a license to display the preprint in perpetuity. It is made available under aCC-BY 4.0 International license.

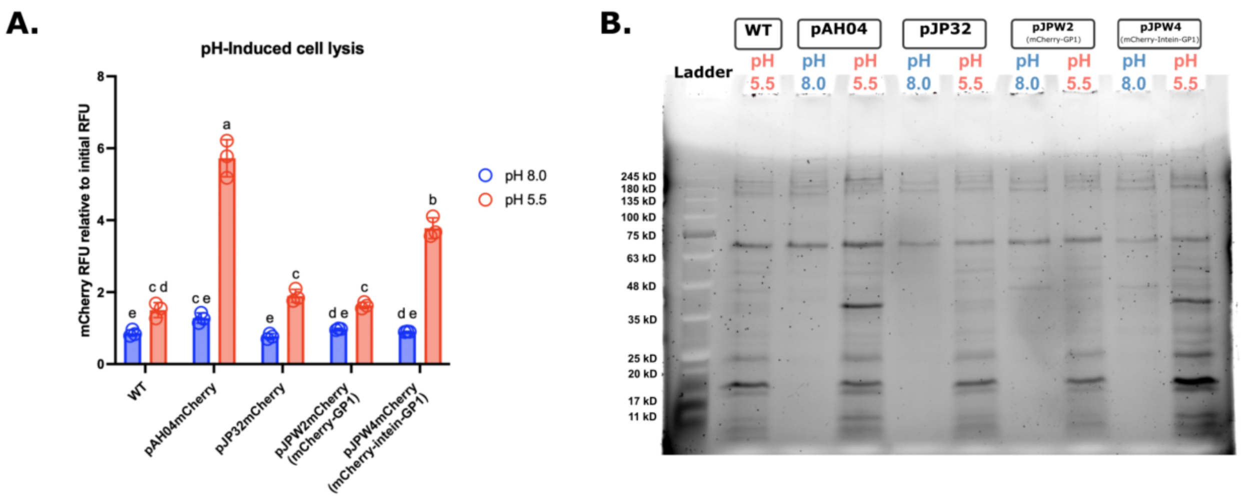

Supplementary Figure 3: Analysis of pH-induced cell lysis in *C. reinhardtii* strains expressing mCherry.

(A) Quantification of mCherry fluorescence released into the supernatant over 2 hours for the strains measured at excitation/emission 580/610 nm. Fluorescence values for the 2-hour time point were normalized to time-zero values and are shown for both pH 8.0 and pH 5.5. Error bars represent the standard deviation of 3 biological replicates. Strains expressing cytosolic mCherry (pAH04mCherry) showed significantly higher mCherry fluorescence release at pH 5.5 compared to pH 8.0, consistent with pH-induced cell lysis. Significant differences between groups are indicated by different letters (e.g., a, b, c, d; $P < 0.05$). **(B)** Stain-free SDS-PAGE gel showing total protein content in the supernatants of *C. reinhardtii* strains cultured in 100mM phosphate buffer at pH 8.0 or 100 mM acetate buffer at pH 5.5 for 2 hours. The lanes include a molecular weight ladder (Ladder) and the following strains: wild type (WT), pAH04mCherry (cytosolic expression of mCherry), pJP32mCherry (secretion of mCherry), mCherry-GP1 (top expressor), and mCherry-intein-GP1 (top expressor). Supernatants from both pH conditions were analyzed for all strains except the WT, which is shown only at pH 5.5 as a control.

bioRxiv preprint doi: https://doi.org/10.1101/2025.01.23.634604; this version posted July 8, 2025. The copyright holder for this preprint (which was not certified by peer review) is the author/funder, who has granted bioRxiv a license to display the preprint in perpetuity. It is made available under aCC-BY 4.0 International license.

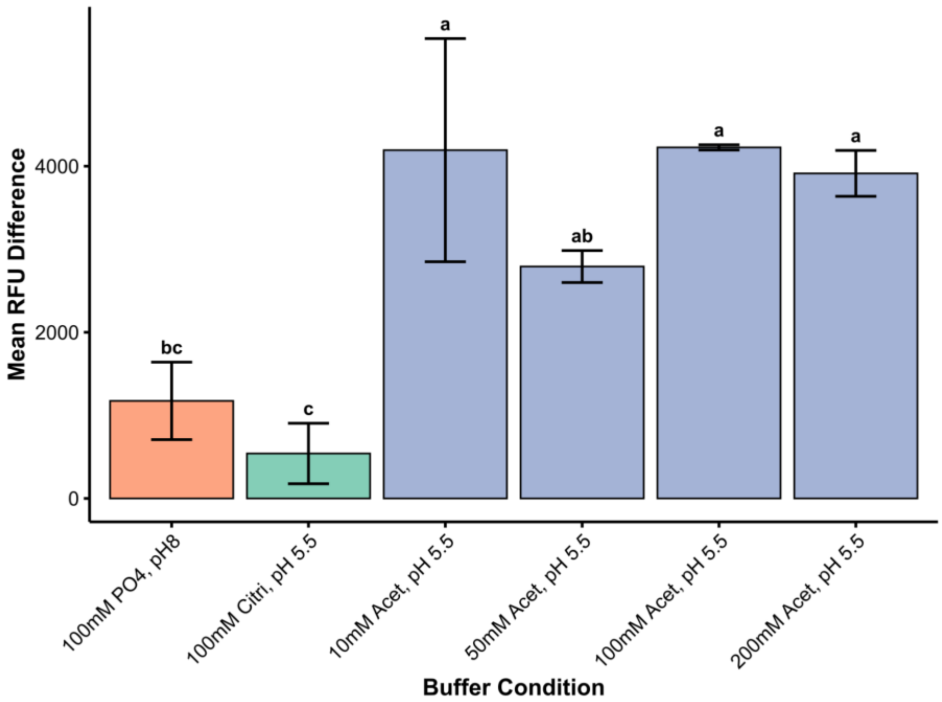

Supplementary Figure 4: Buffer type, pH, and concentration influence cell lysis in *C. reinhardtii* expressing cytosolic mCherry.

Mean mCherry fluorescence release (RFU difference) from the supernatant of *C. reinhardtii* strains expressing cytosolic mCherry after 4 hours under different buffer conditions. Buffers include 100 mM phosphate (PO□) at pH 8.0, 100 mM citrate (Cit) at pH 5.5, and acetate (Acet) at pH 5.5 at concentrations of 10 mM, 50 mM, 100 mM, and 200 mM. Fluorescence readings (Delta RFU) represent mCherry release due to cell lysis, normalized to initial time-zero values. Statistical groupings (letters: a, b, c, etc.) were determined using one-way ANOVA followed by Tukey's post hoc test with three biological replicates per treatment ($P < 0.05$). Error bars represent the standard deviation of replicates.
