# Supplementary material for: Engineering microalgal cell wall-anchored proteins using GP1 PPSPX motifs and releasing with intein-mediated fusion
Source: bioRxiv. 2025 Jan 24:2025.01.23.634604. Preprint. [Version 1] doi: 10.1101/2025.01.23.634604 (PMC11785195; doi:10.1101/2025.01.23.634604)
Supplement: Supplementary file 1 [file media-1.pdf]

Supplementary Figures

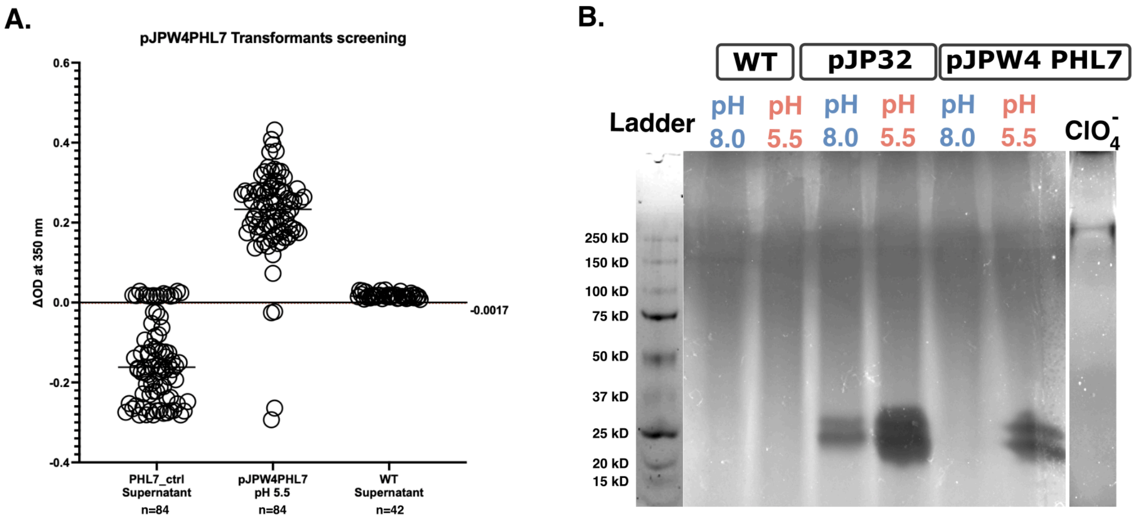

Supplementary Figure 1: Zymogram analysis of plastic-degrading activity in *C.reinhardtii* strains expressing PHL7 under different pH conditions and perchlorate treatment.

(A) Impranil® activity assay to screen for positive pJPW4PHL7 transformants. The threshold (red dotted line) for positive transformants represents three standard deviations below the mean WT supernatant activity (-0.0017). **(B)** This zymogram, performed using Impranil®, a plastic dispersion substrate, demonstrates the proteolytic activity of the plastic-degrading enzyme PHL7 in samples collected from wild type (*WT*), *pJP32* (PHL7-secreting strain), and *pJPW4PHL7* (GP1::Intein::PHL7 fusion protein-expressing strain). The *WT* strain exhibited no detectable activity at either pH 8.0 or pH 5.5. The *pJP32* strain, which secretes PHL7, showed clear proteolytic activity at both pH 8.0 and pH 5.5, indicating functional secretion of the plastic-degrading enzyme. In contrast, the *pJPW4PHL7* strain displayed no detectable activity at pH 8.0, but strong proteolytic activity was observed at pH 5.5, consistent with pH-dependent cleavage and release of the active enzyme from the GP1::Intein::PHL7 fusion construct.

Additionally, treatment of the *pJPW4PHL7* strain with perchlorate (ClO_4^-) resulted in a clearing zone at above 250 kD, indicating an initially uncleaved form of the protein. This result highlights the pH- and chemical-dependent regulation of PHL7 release and activity in engineered *C. reinhardtii* strains.

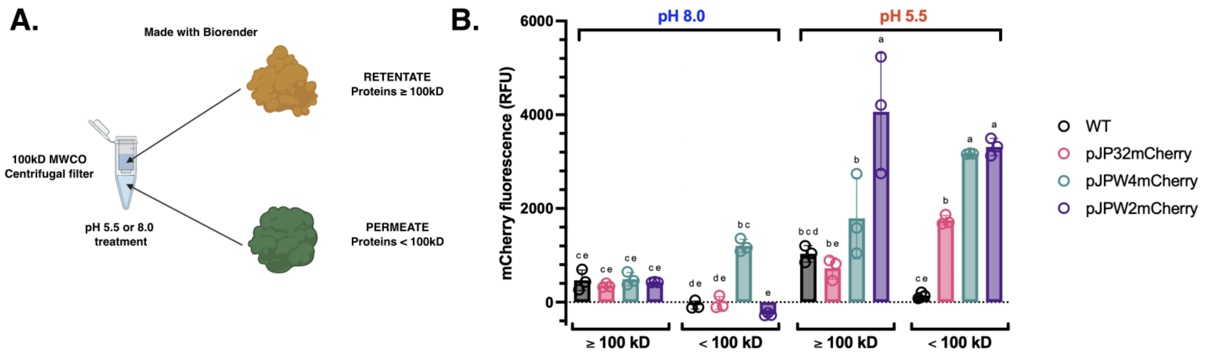

Supplementary Figure 2: Analysis of mCherry fluorescence in supernatants of *C. reinhardtii* strains treated at pH 8.0 and pH 5.5 and filtered through a 100 kDa molecular weight cutoff (MWCO) filter.

Made with Biorender.com

(A) Schematic showing a nanofiltration-based fractionation assay using a 100 kDa molecular weight cutoff filter (MWCO), which allowed us to separate samples into retentate (≥100 kDa) and flowthrough (<100 kDa) fractions. **(B)** The bar plots show mCherry fluorescence, measured in relative fluorescence units (RFU), in two filtrate fractions: proteins smaller than 100 kDa ("<100 kDa") and retained proteins larger than 100 kDa (">100 kDa"). Samples were analyzed from wild type (*Wt*), *pJP32* (secreting non-fused mCherry), *pJPW2* (expressing GP1::mCherry), and *pJPW4* (expressing GP1::pH_Intein::mCherry). **pH 8.0 (left panel)**: Low fluorescence was detected in wild-type, *pJP32 and pJPW2* fractions across both MWCO filters fractions. *pJPW4* strains demonstrated higher fluorescence in the <100 kDa fraction, suggesting partial release of mCherry-containing proteins under neutral conditions. **pH 5.5 (right panel):** Acidic treatment resulted in a substantial increase in fluorescence in the <100 kDa fraction for all recombinant strains, pJP32, *pJPW2* and *pJPW4*, suggesting pH-dependent release of mCherry. *pJP32* exhibited modest fluorescence in the <100 kDa fraction, consistent with secretion of mCherry. The >100 kDa fraction fluorescence decreased significantly, particularly for *pJPW4*, indicating

potential dissociation or cleavage of the fusion protein under acidic conditions. Letters above bars indicate statistical groups, with distinct letters denoting significant differences ($p < 0.05$).

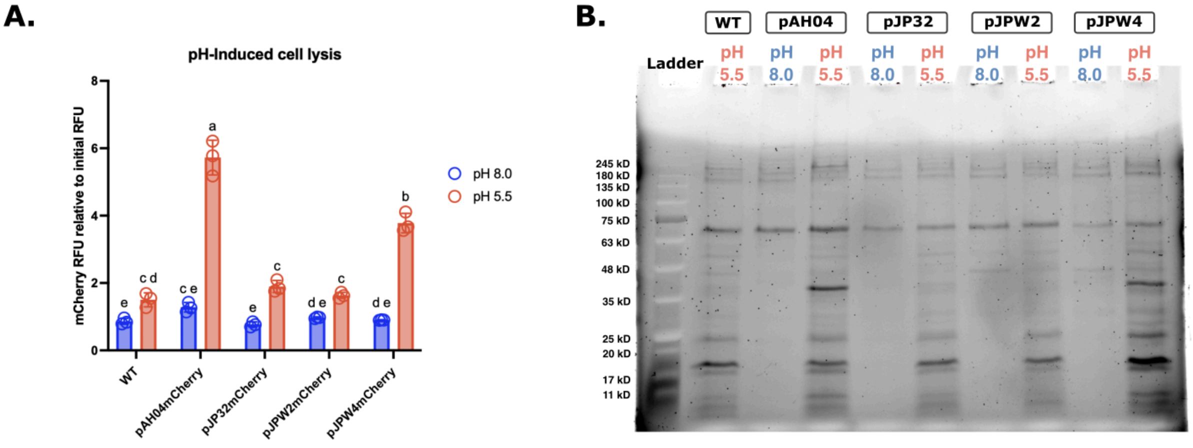

Supplementary Figure 3: Analysis of pH-induced cell lysis in *C. reinhardtii* strains expressing mCherry.

(A) Quantification of mCherry fluorescence released into the supernatant over 2 hours for the strains measured at excitation/emission 580/610 nm. Fluorescence values for the 2-hour time point were normalized to time-zero values and are shown for both pH 8.0 and pH 5.5. Error bars represent the standard deviation of 3 biological replicates. Strains expressing cytosolic mCherry (*pAH04mCherry*) showed significantly higher mCherry fluorescence release at pH 5.5 compared to pH 8.0, consistent with pH-induced cell lysis. Significant differences between groups are indicated by different letters (e.g., a, b, c, d; $P < 0.05$). **(B)** Stain-free SDS-PAGE gel showing total protein content in the supernatants of *Chlamydomonas reinhardtii* strains cultured in 100mM phosphate buffer at pH 8.0 or 100 mM acetate buffer at pH 5.5 for 2 hours. The lanes include a molecular weight ladder (Ladder) and the following strains: wild type (*Wt*), *pAH04mCherry* (cytosolic expression of mCherry), *pJP32mCherry* (secretion of mCherry), *pJPW2mCherry* (cell wall-anchored mCherry via GP1, *mCherry::GP1*), and *pJPW4mCherry* (cell wall-anchored mCherry with an intein linker, *mCherry::intein::GP1*). Supernatants from both pH conditions were analyzed for all strains except the *Wt*, which is shown only at pH 5.5 as a control.

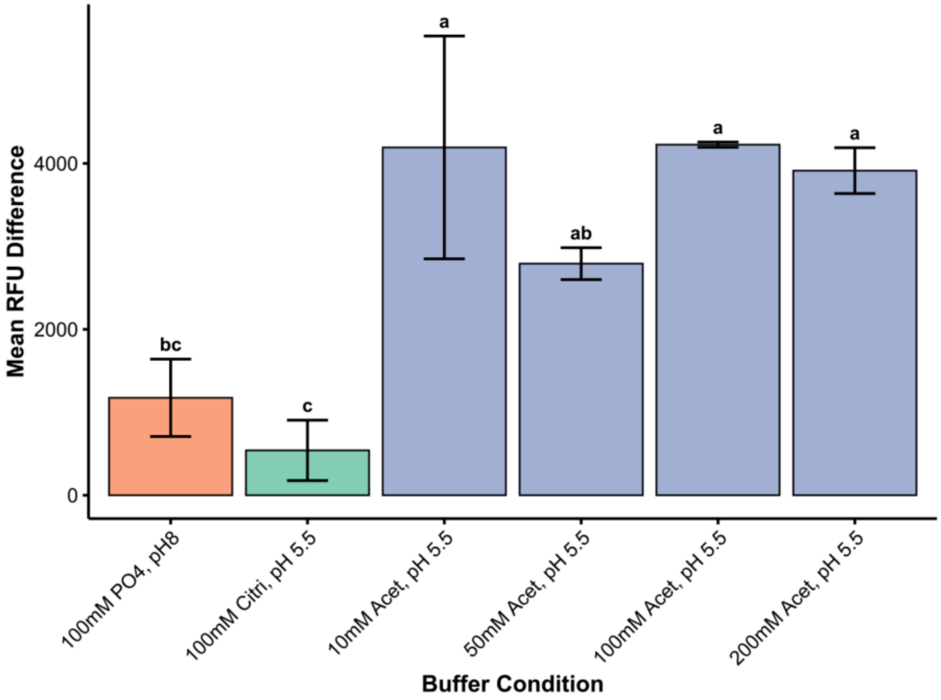

Supplementary Figure 4: Buffer type, pH, and concentration influence cell lysis in *C. reinhardtii* expressing cytosolic mCherry.

Mean mCherry fluorescence release (RFU difference) from the supernatant of *C. reinhardtii* strains expressing cytosolic mCherry after 4 hours under different buffer conditions. Buffers include 100 mM phosphate (PO_4) at pH 8.0, 100 mM citrate (Cit) at pH 5.5, and acetate (Acet) at pH 5.5 at concentrations of 10 mM, 50 mM, 100 mM, and 200 mM. Fluorescence readings (Delta RFU) represent mCherry release due to cell lysis, normalized to initial time-zero values. Statistical groupings (letters: a, b, c, etc.) were determined using one-way ANOVA followed by Tukey's post hoc test with three biological replicates per treatment ($P < 0.05$). Error bars represent the standard deviation of replicates.
